# Supplementary material for: The role of actin protrusion dynamics in cell migration through a degradable viscoelastic extracellular matrix: Insights from a computational model
Source: PLoS Comput Biol. 2020 Jan 13;16(1):e1007250. doi: 10.1371/journal.pcbi.1007250 (PMC6980736; doi:10.1371/journal.pcbi.1007250)
Supplement: S1 Text — (PDF) [file pcbi.1007250.s001.pdf]

S1 Text. Deformable cell model

The cell is modeled as a two dimensional object, consisting of particles connected by viscoelastic (Kelvin-Voigt) elements, that captures the viscoelastic behavior of the membrane and actin cortex and functions as a deformable boundary for the ECM. The stiffness of the actin cortex is modeled as a linear elastic force $\boldsymbol{F}^s$ between connected particles i and j:

$$\boldsymbol{F}_i^s = -k_s \left(\boldsymbol{x}_{ij} - l_{0,ij} \hat{\boldsymbol{e}}_{ij} \right), \tag{1}$$

with k_s the linear spring stiffness (2.8×10^{-3} N/m, assuming a cortex thickness $d_c = 0.4\,\mu$m and Young's modulus $E_c = 6\,$kPa, and with $k_s = \dfrac{2E_c d_c}{\sqrt{3}}$), l_0 the rest length of the spring and

$$\hat{\boldsymbol{e}}_{ij} = \frac{\boldsymbol{x}_i - \boldsymbol{x}_j}{\|\boldsymbol{x}_i - \boldsymbol{x}_j\|} \tag{2}$$

the unit vector from j to i. Dissipation of the membrane and actin cortex is included by the friction term:

$$\boldsymbol{F}_i^{\eta} = -\eta_c \left(\hat{\boldsymbol{e}}_{ij} \cdot \boldsymbol{v}_{ij} \right) \hat{\boldsymbol{e}}_{ij}, \tag{3}$$

with η_c the actin cortex friction.

Bending rigidity is assumed to be weak in order to allow the cell boundary to form sharp protrusions. Therefore, it is used only to prevent the boundary to fold over itself. For internal angles larger than $\theta^* = 225\,^\circ$ the bending moment M is calculated as:

$$M_i = k_{\text{bend}} \left(\theta_i - \theta^* \right) \quad \text{for } \theta_i > \theta^*, \tag{4}$$

with k_{bend} the bending rigidity constant (2×10^{-15} Nm, which is significantly higher than calculated with the formula $k_b = \dfrac{E_c d_c^3}{12\,(1-\nu_c^2)}$, with $\nu_c = 0.45$ the Poisson's ratio of the cortex, in order to ensure that cortex folding is prevented) and θ the internal angle between the two line segments connected to boundary particle i. A corresponding force is applied to boundary particle i and the neighboring boundary particles on either side to restore the boundary angle. An area constraint is used to prevent the cell area from decreasing too much:

$$\boldsymbol{F}_z^A = k_A \left(A^* - A \right) \hat{\boldsymbol{n}}_z \quad \text{for } A < A^*, \tag{5}$$

with A the cell area, A^* the resting cell area, k_A the area constraint constant and $\hat{\boldsymbol{n}}_z$ the outward normal to line segment z that connects neighboring cell boundary particles. Since a too large cell area is already prevented by cortex stiffness and contraction and since protrusions should not be restricted to form, area conservation is applied only when A is smaller than A^* in order to ensure a minimum cell area.

In order to prevent the cell boundary from penetrating itself, a repulsive Hertz-like force is applied to boundary particles that approach a line segment connecting two other neighboring boundary particles of the same cell:

$$\boldsymbol{F}_i^{\text{rep}} = -k_{\text{rep}} \left(d_{\text{rep}} - d_{iz} \right)^{\frac{3}{2}} \hat{\boldsymbol{n}}_z \quad \text{for } d_{iz} < d_{\text{rep}}, \tag{6}$$

with k_{rep} a repulsive force constant, d_{iz} the shortest distance between particle i and line segment z and d_{rep} the particle-line distance below which a repulsive force is applied. An opposite force is

distributed and applied to the boundary particles connected by the line segment. Finally, as fluid ECM particles do not interact with the cell boundary, an ECM particle-independent drag force $\boldsymbol{F}^d$ due to interaction with the medium is applied:

$$\boldsymbol{F}_i^d = -\gamma_{liquid}\boldsymbol{v}_i, \tag{7}$$

with γ_{liquid} the liquid drag force constant for contact with the medium.
