# Supplementary material for: The role of actin protrusion dynamics in cell migration through a degradable viscoelastic extracellular matrix: Insights from a computational model
Source: PLoS Comput Biol. 2020 Jan 13;16(1):e1007250. doi: 10.1371/journal.pcbi.1007250 (PMC6980736; doi:10.1371/journal.pcbi.1007250)
Supplement: S2 Text — (PDF) [file pcbi.1007250.s002.pdf]

S2 Text. SPH implementation of the ECM model

As described before [1], the conservation of momentum for a viscoelastic material modeled by SPH is implemented as:

$$m_i \frac{\mathrm{d}\boldsymbol{v}_i}{\mathrm{d}t} = \boldsymbol{F}_i^\sigma + \boldsymbol{F}_i^v + \boldsymbol{F}_i^b, \tag{1}$$

with $\boldsymbol{F}^\sigma$ the elastic forces, $\boldsymbol{F}^v$ the viscous forces and $\boldsymbol{F}^b$ body forces. The elastic forces between particle i and its neighboring particles j within a smoothing kernel are calculated as:

$$\boldsymbol{F}_i^{\sigma\,\alpha} = m_i \sum_j m_j \left(\frac{\sigma_i^{\alpha\beta}}{\rho_i^2} + \frac{\sigma_j^{\alpha\beta}}{\rho_j^2} \right) \boldsymbol{\nabla}_i^\beta W_{ij}, \tag{2}$$

with $\boldsymbol{\sigma}$ the stress tensor and $\boldsymbol{\nabla}_i W_{ij}$ the derivative of the smoothing kernel W. The stress tensor $\boldsymbol{\sigma}$ for an elastic solid consists of the hydrostatic and deviatoric stress:

$$\sigma_i^{\alpha\beta} = -p_i \delta^{\alpha\beta} + S_i^{\alpha\beta}, \tag{3}$$

written in Einstein notation with respect to the coordinate indices α and β, with $\boldsymbol{\delta}$ denoting the Kronecker delta, p the hydrostatic pressure and $\boldsymbol{S}$ the deviatoric stress. The hydrostatic pressure is calculated using the equation of state that defines the relationship between pressure and density of a particle:

$$p_i = p_0 + K \left(\left(\frac{\rho_i}{\rho_0} \right) - 1 \right), \tag{4}$$

where ρ_0 and p_0 are the initial density and pressure and K is the bulk modulus ($K = \frac{E}{3(1-2\nu)}$, with E the Young's modulus and ν the Poisson's ratio) [2]. The deviatoric stress according to Hooke's law reads:

$$S^{\alpha\beta} = 2G \left(\epsilon^{\alpha\beta} - \frac{1}{3} \delta^{\alpha\beta} \epsilon^{\gamma\gamma} \right), \tag{5}$$

with G the shear modulus ($G = \frac{E}{2(1+\nu)}$) and $\boldsymbol{\epsilon}$ the strain tensor. The Jaumann rate of change of the deviatoric stress is then calculated by:

$$\frac{\mathrm{d}S_i^{\alpha\beta}}{\mathrm{d}t} = 2G \left(\dot{\epsilon}_i^{\alpha\beta} - \frac{1}{3} \delta^{\gamma\gamma} \dot{\epsilon}_i^{\alpha\beta} \right) + S_i^{\alpha\beta} \Omega_i^{\beta\gamma} + \Omega_i^{\alpha\gamma} S_i^{\gamma\beta}, \tag{6}$$

with $\dot{\boldsymbol{\epsilon}}$ the strain rate tensor:

$$\dot{\epsilon}^{\alpha\beta} = \frac{1}{2} \left(\frac{\partial v^\alpha}{\partial x^\beta} + \frac{\partial v^\beta}{\partial x^\alpha} \right) \tag{7}$$

and $\boldsymbol{\Omega}$ the spin tensor:

$$\Omega^{\alpha\beta} = \frac{1}{2} \left(\frac{\partial v^\alpha}{\partial x^\beta} - \frac{\partial v^\beta}{\partial x^\alpha} \right). \tag{8}$$

The viscous forces are calculated according to the formulation of Morris *et al.* [3]:

$$\boldsymbol{F}_i^v = m_i \sum_j m_j \frac{\mu_i + \mu_j}{\rho_i \rho_j} \frac{\boldsymbol{x}_{ij} \cdot \boldsymbol{\nabla}_i W_{ij}}{\mid \boldsymbol{x}_{ij} \mid^2 + \eta^2} \boldsymbol{v}_{ij}, \tag{9}$$

with μ the dynamic viscosity of the fluid and $\eta = 0.01h^2$ a correction factor that prevents singularity when particles approach each other.

As the processes described by the model occur at low Reynolds number, inertial forces in the conservation of momentum can be omitted. This results in the following equation:

$$-m_i \sum_j m_j \frac{\mu_i + \mu_j}{\rho_i \rho_j} \frac{\boldsymbol{x}_{ij} \cdot \boldsymbol{\nabla}_i W_{ij}}{\mid \boldsymbol{x}_{ij} \mid^2 + \eta^2} \boldsymbol{v}_{ij} = m_i \sum_j m_j \left(\frac{\boldsymbol{\sigma}_i}{\rho_i^2} + \frac{\boldsymbol{\sigma}_j}{\rho_j^2} \right) \cdot \boldsymbol{\nabla}_i W_{ij} + \boldsymbol{F}_i^b. \tag{10}$$

As Van Liedekerke *et al.* showed, the left-hand side of this equation can be rewritten by introducing a friction matrix Γ and by assuming that $m_i = m_j$ [4]:

$$\sum_j \Gamma_{ij} \boldsymbol{v}_{ij} = \sum_j \left(\boldsymbol{\sigma}_i V_i^2 + \boldsymbol{\sigma}_j V_j^2 \right) \cdot \boldsymbol{\nabla}_i W_{ij} + \boldsymbol{F}_i^b, \tag{11}$$

with $V_i = \dfrac{m_i}{\rho_i}$ the particle volume and with the friction matrix:

$$\Gamma_{ij} = (\mu_i + \mu_j) V_i V_j \frac{\boldsymbol{x}_{ij} \cdot \boldsymbol{\nabla}_i W_{ij}}{\mid \boldsymbol{x}_{ij} \mid^2 + \eta^2}. \tag{12}$$

This equation is solved with a Conjugate Gradient Method [5] and directly provides the velocities of the particles instead of accelerations. Van Liedekerke *et al.* demonstrated that NSPH is able to solve creeping flow problems with a time step of up to three orders of magnitude higher than SPH [4].

References

[1] T. Heck, B. Smeets, S. Vanmaercke, P. Bhattacharya, T. Odenthal, H. Ramon, H. Van Oosterwyck, P. Van Liedekerke, Modeling extracellular matrix viscoelasticity using smoothed particle hydrodynamics with improved boundary treatment, Computer Methods in Applied Mechanics and Engineering 322 (2017) 515–540. `doi:10.1016/j.cma.2017.04.031`.

[2] J. Monaghan, Simulating free surface flows with SPH, Journal of Computational Physics 110 (1994) 399–406.

[3] J. P. Morris, P. J. Fox, Y. Zhu, Modeling low Reynolds number incompressible flows using SPH, Journal of Computational Physics 136 (1) (1997) 214–226. `doi:10.1006/jcph.1997.5776`.
URL `http://linkinghub.elsevier.com/retrieve/pii/S0021999197957764`

[4] P. Van Liedekerke, B. Smeets, T. Odenthal, E. Tijskens, H. Ramon, Solving microscopic flow problems using Stokes equations in SPH, Computer Physics Communications 184 (7) (2013) 1686–1696. `doi:10.1016/j.cpc.2013.02.013`.
URL `http://linkinghub.elsevier.com/retrieve/pii/S0010465513000702`

[5] M. T. Heath, Scientific computing: an introductory survey, 2nd Edition, McGraw-Hill, 2005.
