# Supplementary material for: The role of actin protrusion dynamics in cell migration through a degradable viscoelastic extracellular matrix: Insights from a computational model
Source: PLoS Comput Biol. 2020 Jan 13;16(1):e1007250. doi: 10.1371/journal.pcbi.1007250 (PMC6980736; doi:10.1371/journal.pcbi.1007250)
Supplement: S3 Text — (PDF) [file pcbi.1007250.s003.pdf]

S3 Text. ECM degradation and cell-ECM boundary conditions

ECM degradation is modeled by gradual fluidization of ECM particles. Degradation occurs at the protrusion tip and close to the cell body and is implemented by a degradation factor f_{degr}, which has a value between 1 (intact solid ECM) and 0 (fully degraded ECM). In order to prevent hindrance of the cell by degraded material, the SPH formulation and boundary conditions are adapted such that the degraded ECM particles are allowed to move freely through the cell. This requires to divide the ECM particles into two phases for which different formulations apply: a solid (s) intact ECM phase ($f_{\mathrm{degr}} > 0$) and a fluid (f) degraded ECM phase ($f_{\mathrm{degr}} = 0$). In order to clearly distinguish the solid and fluid phase and to prevent formation of very weak solid particles, f_{degr} is set to 0 as soon as it drops below 0.1.

When a cell pushes against the ECM, the solid ECM deforms and resists cell movement by buildup of hydrostatic pressure and deviatoric stress. Fluid particles are allowed to move freely through the cell boundary and therefore do not build up hydrostatic pressure due to contact with the cell. However, fluid particles can still build up hydrostatic pressure due to contact with neighboring ECM particles. If a cell boundary pushes against solid ECM particles, these particles could lower their hydrostatic pressure by pushing away neighboring fluid particles within the cell, resulting in a reduced cell-ECM contact force and an inability of the solid ECM to resist cell migration. In order to prevent his, fluid and solid mechanics are separated in the SPH formulation by giving the ECM particles two densities: a fluid density ρ_f that is affected by fluid-fluid and fluid-solid particle contact and a solid density ρ_s that is affected by solid-solid and solid-boundary particle contact. The change in fluid density is calculated as:

$$\frac{\mathrm{d}\rho_{f,i}}{\mathrm{d}t} = \sum_{j \in \mathcal{F} \cup \mathcal{S} | (i \in \mathcal{F} \vee j \in \mathcal{F})} m_j \boldsymbol{v}_{ij} \cdot \boldsymbol{\nabla}_i W_{ij}, \tag{1}$$

with $\mathcal{F}$ the set of fluid ECM particles and $\mathcal{S}$ the set of solid ECM particles. The solid density ρ_s is affected by solid-solid and solid-boundary particle contact:

$$\frac{\mathrm{d}\rho_{s,i}}{\mathrm{d}t} = \frac{1}{\gamma_i} \sum_{j \in \mathcal{S} \cup \mathcal{B}} m_j \boldsymbol{v}_{ij} \cdot \boldsymbol{\nabla} W_{ij} - \frac{\rho_{s,i}}{\gamma_i} \sum_{z \in \mathcal{Z}} \mathbf{v}_{iz} \cdot \boldsymbol{\nabla} \gamma_{iz}, \tag{2}$$

with $\mathcal{B}$ the set of boundary particles, $\mathcal{Z}$ the set of line segments connecting the boundary particles and γ and $\boldsymbol{\nabla}\gamma$ the renormalization factor and gradient of the renormalization factor. This renormalization factor is introduced to correct the SPH formulations for an incomplete kernel support at the cell boundary as introduced before [1, 2, 3]. A change in ρ_f and ρ_s results in a hydrostatic pressure p_f that leads to pressure-related forces for fluid-fluid and fluid-solid particle contact and a hydrostatic pressure p_s that leads to pressure-related forces for solid-solid and solid-boundary particle contact. With this implementation hydrostatic pressure that is built up by cell-ECM contact cannot be relieved by pushing away fluid particles, while total hydrostatic pressure buildup in the ECM according to the standard SPH formulation is respected. When a particle is degraded (by decreasing f_{degr}) it gradually changes from a solid to a fluid phase. This means that the change in ρ_s that accumulated in all earlier time steps for solid-solid particle contacts is converted in a change in ρ_f as the particle is being degraded. Besides, the accumulated change in deviatoric stress S is gradually decreased as the particle is degraded. In this way no residual changes in ρ_s and S remain for solid particles neighboring the fully degraded particle. The accumulated change in ρ_s and S for solid particles due to contact with the cell boundary is subtracted only when f_{degr} drops below the

chosen solid-fluid transition value 0.1. In this way penetration of the cell boundary by particles that are transitioning from the solid to the fluid phase is prevented. A free slip boundary condition is ensured by only allowing build up of normal stress at the boundary and only allowing contact forces in the direction normal to the boundary.

Modeling of contact between the ECM and a deformable cell requires adaptation compared to contact with a rigid boundary as described in [1]. First, the density of the boundary particles should be equal to the ECM particles and the volume of the boundary particles is defined by the angle between the two connected line segments. Therefore, the mass of a boundary particle changes with boundary curvature, resolution and stretching and is calculated as:

$$m_i = \frac{l_{z,i}}{dp} m_0 \frac{\theta}{2\pi}, \tag{3}$$

with $l_{z,i}$ the average length of the two line segments connected to the boundary particle, dp the typical particle distance in the ECM, $m_0 = \rho_0 dp^2$ the reference mass for an initial density ρ_0 ($1000\,\mathrm{kg/m^3}$) and θ the external angle between the two line segments connected to the boundary particle.

References

[1] T. Heck, B. Smeets, S. Vanmaercke, P. Bhattacharya, T. Odenthal, H. Ramon, H. Van Oosterwyck, P. Van Liedekerke, Modeling extracellular matrix viscoelasticity using smoothed particle hydrodynamics with improved boundary treatment, Computer Methods in Applied Mechanics and Engineering 322 (2017) 515–540. doi:10.1016/j.cma.2017.04.031.

[2] S. Kulasegaram, J. Bonet, R. W. Lewis, M. Profit, A variational formulation based contact algorithm for rigid boundaries in two-dimensional SPH applications, Computational Mechanics 33 (4) (2004) 316–325. doi:10.1007/s00466-003-0534-0.

[3] M. Ferrand, D. R. Laurence, B. D. Rogers, D. Violeau, C. Kassiotis, Unified semi-analytical wall boundary conditions for inviscid, laminar or turbulant flows in the meshless SPH method, International Journal for numerical methods in fluids 71 (2013) 446–472.
