# Supplementary material for: The role of actin protrusion dynamics in cell migration through a degradable viscoelastic extracellular matrix: Insights from a computational model
Source: PLoS Comput Biol. 2020 Jan 13;16(1):e1007250. doi: 10.1371/journal.pcbi.1007250 (PMC6980736; doi:10.1371/journal.pcbi.1007250)
Supplement: S4 Text — (PDF) [file pcbi.1007250.s004.pdf]

S4 Text. Protrusion deflection

Since the protrusion growth direction $\boldsymbol{d}_{\text{prot}}$ is constant, a protrusion will always form in this direction independent of the local solid ECM. However, in theory the ECM could function as an obstacle and deflect the protrusion such that it grows in a direction with less solid ECM hindrance. This deflection of the protrusion growth direction is modeled by rotating $\boldsymbol{d}_{\text{prot}}$ away from $\boldsymbol{d}_{\perp\text{ECM}}$, a vector directed perpendicular to the local solid ECM around the protrusion tip, with a rotation rate $\frac{\mathrm{d}\theta_{\text{prot}}}{\mathrm{d}t}$:

$$\frac{\mathrm{d}\theta_{\text{prot}}}{\mathrm{d}t} = r_{\text{defl}} \frac{\|\boldsymbol{d}_{\perp\text{ECM}}\|}{h} \left(1 - \cos\left(\theta\right)\right) \tag{1}$$

towards $\boldsymbol{d}_{\|\text{ECM}}$, a vector parallel to the local solid ECM closest to $\boldsymbol{d}_{\text{prot}}$ (see Fig A). r_{defl} is a reference deflection rate, $h = 1.3dp$ the smoothing length and θ the angle between $\boldsymbol{d}_{\|\text{ECM}}$ and $\boldsymbol{d}_{\text{prot}}$. $\boldsymbol{d}_{\perp\text{ECM}}$ is calculated as the weighted average direction of the solid particles around the tip protrusion particle i using the smoothing kernel:

$$\boldsymbol{d}_{i,\perp\text{ECM}} = -\sum_{j\in\mathcal{S}} \frac{m_j}{\rho_j} \boldsymbol{x}_{ij} W_{ij}. \tag{2}$$

In this way a protrusion deflects faster when it is closer to the ECM and when it is more parallel to the solid ECM surface. A protrusion that initially grows perpendicular to the ECM will not deflect fast enough and still degrade and grow into the ECM. When a protrusion deflects from the ECM, formation of an adhesion in the protrusion direction could place the adhesion in a degraded part of the ECM. In order to prevent this, the adhesion is formed in the direction of $\boldsymbol{d}_{\perp\text{ECM}}$ when the angle between $\boldsymbol{d}_{\text{prot}}$ and $\boldsymbol{d}_{\perp\text{ECM}}$ is larger than $30\,^\circ$.

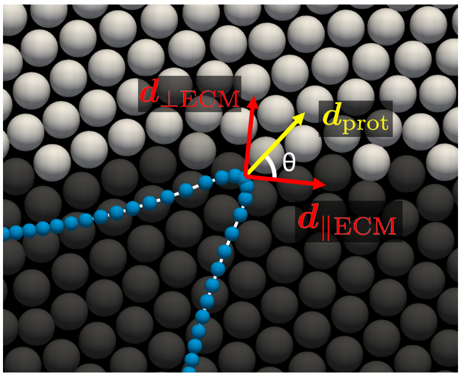

Fig A: The protrusion growth direction $\boldsymbol{d}_{\text{prot}}$ rotates away from the ECM direction $\boldsymbol{d}_{\perp\text{ECM}}$ towards the direction parallel to the local ECM $\boldsymbol{d}_{\|\text{ECM}}$.
