# Supplementary material for: The role of actin protrusion dynamics in cell migration through a degradable viscoelastic extracellular matrix: Insights from a computational model
Source: PLoS Comput Biol. 2020 Jan 13;16(1):e1007250. doi: 10.1371/journal.pcbi.1007250 (PMC6980736; doi:10.1371/journal.pcbi.1007250)
Supplement: S5 Text — (PDF) [file pcbi.1007250.s005.pdf]

# S5 Text. Cortex curvature-dependent actomyosin contraction

The effect of cortex curvature on contraction is captured by a curvature factor $f_{\mathrm{curv}}$. $f_{\mathrm{curv}}$ as function of the average curvature of two particles connected to a line segment is shown in Fig B and calculated as:

$$f_{\mathrm{curv},ij} = \begin{cases} 0.6e^{-\dfrac{(\kappa_{ij}-\kappa_0)^2}{2\kappa_w^2}} & \text{for } |\kappa_{ij}-\kappa_0| \leq \kappa_w \ , \\ 0 & \text{for } |\kappa_{ij}-\kappa_0| > \kappa_w \end{cases} \quad (1)$$

with $\kappa_{ij} = \dfrac{\kappa_i + \kappa_j}{2}$ the average curvature $\kappa$ of particles $i$ and $j$, $\kappa_0$ = -0.15 the optimal cortex curvature for myosin II binding and $\kappa_w = 0.25$ the range of curvature values around $\kappa_0$ for which myosin II binding is possible (see Fig B). $\kappa$ is calculated as the Menger curvature of a particle and the two neighboring particles. It should be noted that Elliott *et al.* correlated myosin II intensity to mean curvature, which is the average of the two principal curvatures in 3D [1]. For a cell plated into a microfabricated agarose chamber, at the walls one principal curvature is always positive, while the second principal curvature depends on the local curvature of the chamber. Therefore, localization of myosin II at regions of minimal mean curvature in 3D coincides with localization of myosin II at regions with slight negative curvature in a 2D model. The maximal value of $f_{\mathrm{curv}}$ is set to 0.6 in order to generate a similar contractile force on the adhesion during and after protrusion maturation. After maturation the actomyosin contractile force $\boldsymbol{F}^{\mathrm{mat}}$ is replaced by a contractile force $\boldsymbol{F}^{\mathrm{am}}$ on all line segments of the protrusion, which at optimal curvature would generate a contractile force almost twice as high as $\boldsymbol{F}^{\mathrm{mat}}$ (because a protrusion consists of two series of springs attached to the adhesion particle) and would therefore result in direct adhesion rupture. This is prevented by reducing the maximal value of $f_{\mathrm{curv}}$ to a maximal value of 0.6.

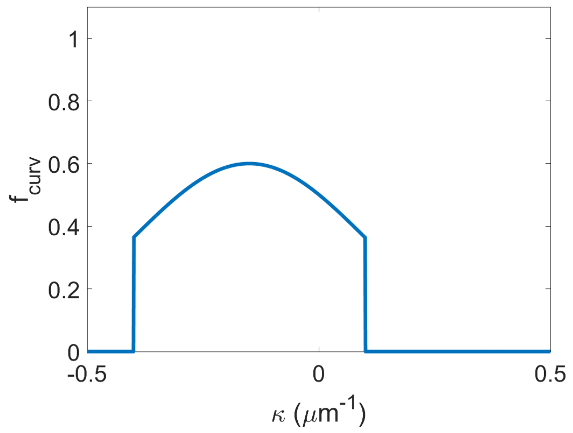


Fig B: Curvature-dependent ($\kappa$) association of myosin II to the actin cortex is captured by $f_{\mathrm{curv}}$.

## References


[1] H. Elliott, R. S. Fischer, K. A. Myers, R. A. Desai, L. Gao, C. S. Chen, R. S. Adelstein, C. M. Waterman, G. Danuser, Myosin II controls cellular branching morphogenesis and migration in

three dimensions by minimizing cell-surface curvature, Nature Cell Biology 17 (2) (2015) 137–147. arXiv:15334406, doi:10.1038/ncb3092.
