# Supplementary material for: The role of actin protrusion dynamics in cell migration through a degradable viscoelastic extracellular matrix: Insights from a computational model
Source: PLoS Comput Biol. 2020 Jan 13;16(1):e1007250. doi: 10.1371/journal.pcbi.1007250 (PMC6980736; doi:10.1371/journal.pcbi.1007250)
Supplement: S1 Fig — (PDF) [file pcbi.1007250.s006.pdf]

S1 Figure. Cell Polarization

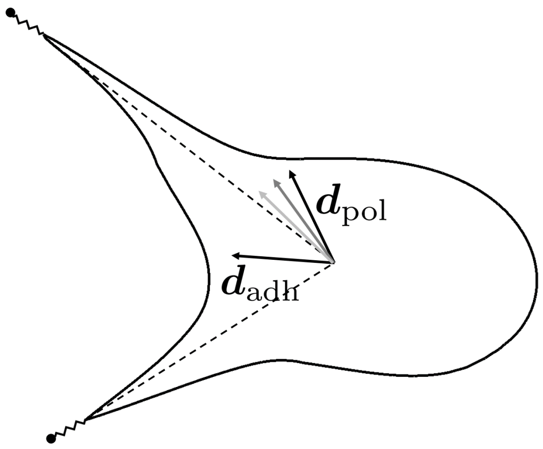

Figure 1: Illustration of polarization of the cell towards the average adhesion direction. The polarization direction $\boldsymbol{d}_{\mathrm{pol}}$ rotates with a fixed polarization rate r_{pol} towards the average adhesion direction $\boldsymbol{d}_{\mathrm{adh}}$ as seen from the center of mass of the cell. When the polarization direction reaches the average adhesion direction it stops rotating until the average adhesion direction is changed.
