# Supplementary material for: The role of actin protrusion dynamics in cell migration through a degradable viscoelastic extracellular matrix: Insights from a computational model
Source: PLoS Comput Biol. 2020 Jan 13;16(1):e1007250. doi: 10.1371/journal.pcbi.1007250 (PMC6980736; doi:10.1371/journal.pcbi.1007250)
Supplement: S2 Fig — (PDF) [file pcbi.1007250.s007.pdf]

S2 Figure. Simulation readout correlation

E_ecm	v_migr_path	l_prot	n_prot	t_prot
v_migr_abs	0.586	0.621	0.461	0.341
v_migr_path		0.571	0.822	0.723
l_prot			0.526	0.432
n_prot				0.787

F_am	v_migr_path	l_prot	n_prot	t_prot
v_migr_abs	0.228	0.157	0.296	0.0176
v_migr_path			0.622	0.470
l_prot				
n_prot				0.580

P_prot	v_migr_path	l_prot	n_prot	t_prot
v_migr_abs		0.131		
v_migr_path		0.185	0.555	
l_prot			0.149	0.075
n_prot				

P_off_min	v_migr_path	l_prot	n_prot	t_prot
v_migr_abs		0.489		
v_migr_path		0.091	0.356	0.206
l_prot				
n_prot				0.667

T_prot	v_migr_path	l_prot	n_prot	t_prot
v_migr_abs				
v_migr_path		0.438		
l_prot			0.225	0.108
n_prot				0.550

E_ecm, ζ=8e4	v_migr_path	l_prot	n_prot	t_prot
v_migr_abs	0.348	0.581	0.190	0.176
v_migr_path		0.224	0.677	0.452
l_prot			0.121	0.107
n_prot				0.666

E_ecm, P_off	v_migr_path	l_prot	n_prot	t_prot
v_migr_abs	0.422	0.666	0.081	
v_migr_path		0.457	0.188	
l_prot			0.289	
n_prot				0.356

	p<=0.005	p<=0.01	p<=0.05	p>0.05
increase				
decrease				

Figure 1: Simulation readout correlation for 7 sets of simulations in which a single one of the following 7 parameters is varied: ECM stiffness (E_{ECM}) with force-dependent adhesion disassembly ($\zeta_{\mathrm{diss}} = 2\times10^4$), cell strength (reference actomyosin contractile force F_{am}), total number of protrusions (protrusion initiation rate r_{prot}), average protrusion lifetime (adhesion disassembly rate at normal load $r_{\mathrm{off,min}}$), average protrusion length (protrusion growth time T_{prot}), ECM stiffness (E_{ECM}) with force-dependent adhesion disassembly ($\zeta_{\mathrm{diss}} = 8\times10^4$) and ECM stiffness ($E_{\mathrm{ECM}}$) with force-independent adhesion disassembly. Coefficients of determination (R^2-values) are calculated for each combination of 5 simulation readouts: absolute migration velocity $v_{\mathrm{migr,abs}}$, migration velocity along cell path $v_{\mathrm{migr,path}}$, average protrusion length l_{prot}, total number of protrusions n_{prot} and average protrusion lifetime τ_{prot}. Color represents significance (brightness according to p-value) of positive (green) or negative (red) correlation.
