# Supplementary material for: The role of actin protrusion dynamics in cell migration through a degradable viscoelastic extracellular matrix: Insights from a computational model
Source: PLoS Comput Biol. 2020 Jan 13;16(1):e1007250. doi: 10.1371/journal.pcbi.1007250 (PMC6980736; doi:10.1371/journal.pcbi.1007250)
Supplement: Attachment — Submitted filename: Review of Manuscript PCOMPBIOL-D-19-01125.pdf [file pcbi.1007250.s012.pdf]

Review of Manuscript PCOMPBIOL-D-19-01125

The role of actin protrusion dynamics in cell migration through a degradable viscoelastic extracellular matrix: Insights from a computational model

Authors: *Tommy Heck, Diego Arango Vargas, Bart Smeets, Herman Ramon, Paul Van Liedekerke and Hans Van Oosterwyck*.

The authors present a very interesting and mathematically rigorous computational model of a two-dimensional representation of the cell migrating through a three-dimensional extracellular matrix. The methodology presented is novel and could potentially be used for further simulation and study of cellular interaction with 3D ECMs. Furthermore, the computational model is formulated on a variety of experimental studies. The paper is well-written and provides insight on how protrusion numbers and dynamics affects motility and interaction with the ECM.

Major comments:

1. In the abstract and introduction it should be mentioned that the authors are modeling a two-dimensional representation of a cell migrating through a 3D extracellular matrix. This is mentioned later in the manuscript, and as it stands now, is misleading to readers.

2. Page 10, line 190: The authors mention that a strain-dependent Young's modulus can be used to incorporate strain stiffening into the model. This is the only time a Young's modulus is mentioned in the entire manuscript. A constitutive equation relating elastic stresses to strains should be specified in Supplement S2.

3. Section Protrusion Dynamics: The introductory paragraph in this section provides a nice overview. However, as one is reading the subsections, it is not clear to the reader that protrusion growth and maturation each occur for a fixed, predetermined amount of time. It is an important assumption of the model that protrusion lifetime is determined only from the adhesion disassembly rate, and it should be made clear early in this section that this is the assumption and that the rates of growth and maturation are fixed. Also, a justification/explanation of *why* these assumptions are made, specifically why growth and maturations rates are fixed, should be provided earlier in the manuscript rather than only at the end of the discussion section.

4. Page 19, line 418-419: Here the absolute migration velocity is described. Is this the best way to measure *velocity*? The term velocity is typically understood as the rate of change of the position along a path, and the authors do calculate a *path velocity* later in the results section of the manuscript. An explanation of why the authors use an absolute migration velocity instead of the path velocity is needed. Is this velocity used in experimental results? If so, a reference should be given. If this choice of velocity is not based on experimental results a justification to its use should be provided.

5. Following up on comment above: The manuscript would benefit from validating a portion of the model by comparison to experimental results. One suggestion would be to simply reference experimental results which verify that measured absolute migration velocities (or cell path velocities) compare to the numerical results. A stronger comparison would be to compare experimentally measured absolute migration velocities (or, again, cell path velocities – whatever is more appropriate given the experimental set up) for different substrate stiffnesses to the numerical results in Figure 7.

6. Page 25, lines 569-571: The first two sentences here are confusing and seem to contradict one another. They need to be reworded so the point author is attempting to make is clearer.

7. Figure 11 and last sentence on paragraph p. 28 lines 651-653: Figure 11 is not particularly enlightening as is. Possibly, instead of displacements, colors can correspond to stresses since the applied stresses are a measure of the maturity of the adhesion. The idea is to better illustrate that protrusions in the direction of the fibers are more mature than adhesions perpendicular to the fibers. As it stands currently, this figure does not do much to support the claim on lines 651-653.

8. Page 30, lines 689-691: There is no evidence in the paper to support the claim "In our model, varying protrusion number, lifetime and length did not affect both the average absolute migration velocity and ***average migration velocity along the cell path*** ". The average migration path velocity is only measured when number of protrusions is varied (not lifetime and length of protrusion). This sentence should be modified to reflect what is supported with results.

Minor comments:

9. The work most similar to the manuscript is what has been published in reference [11]. However, a major aim of the work in reference [11] is to determine the effective cell-probed stiffness of the ECM, which is entirely different than the aim of the submitted manuscript. The authors should highlight this as it will strengthen the novel contribution they are making with the manuscript.
10. Figure 4 caption: Parenthetical should state "(see Eq 7 *and surrounding text* for parameter meanings)."
11. Figure 7 is difficult to read. It should be broken up into two figures. One option is to put ECM stiffness and cell strength variations in one figure and adhesion initiation rate, disassembly rate, and growth time in another figure.
12. In Figures 7-10, an explanation of the horizontal bars at the top of each panel is needed.
13. Figure 8 is also difficult to read. Break up into two figures. Part (A) can go into one figure while parts (B)-(D) can go into another.

14. Figure 9 caption and Figure 10 caption: "Time lag" needs to be defined.

15. Page 30, line 693-695: Looking at Figure 8 B-D, visually, there is a difference in average migration velocities between force dependent and independent adhesion disassembly. Rather than stating that no difference in migration velocity was observed, it would be better to state the differences in migration velocity are not statistically significant.

16. S1 Text, line immediately after equation (2): should be unit vector from i to j (not j to i, as stated).

17. S1 Text, lines immediately after equations (5) and (6): "line segment z" needs to be defined.
