# Supplementary material for: The role of actin protrusion dynamics in cell migration through a degradable viscoelastic extracellular matrix: Insights from a computational model
Source: PLoS Comput Biol. 2020 Jan 13;16(1):e1007250. doi: 10.1371/journal.pcbi.1007250 (PMC6980736; doi:10.1371/journal.pcbi.1007250)
Supplement: Attachment — Submitted filename: response_to_reviewers.pdf [file pcbi.1007250.s013.pdf]

We would like to thank the editor for the opportunity to submit a revised version of the manuscript, and the reviewers for their comments and suggestions, that helped us to improve the quality of the manuscript. The text has been revised and rewritten in some parts. All the changes are highlighted in blue in the new version of the manuscript. Please find below our response to the reviewer's comments, their original comments mentioned in black.

Reviewer 1

The role of actin protrusion dynamics in cell migration through a degradable viscoelastic extracellular matrix: Insights from a computational model

Authors: *Tommy Heck, Diego Arango Vargas, Bart Smeets, Herman Ramon, Paul Van Liedekerke and Hans Van Oosterwyck*.

The authors present a very interesting and mathematically rigorous computational model of a two-dimensional representation of the cell migrating through a three-dimensional extracellular matrix. The methodology presented is novel and could potentially be used for further simulation and study of cellular interaction with 3D ECMs. Furthermore, the computational model is formulated on a variety of experimental studies. The paper is well-written and provides insight on how protrusion numbers and dynamics affects motility and interaction with the ECM.

We are grateful for the appreciation of the work by the reviewer.

Major comments:

1. In the abstract and introduction it should be mentioned that the authors are modeling a two-dimensional representation of a cell migrating through a 3D extracellular matrix. This is mentioned later in the manuscript, and as it stands now, is misleading to readers.

The fact that the model is a 2D representation of 3D cell migration is now mentioned in both the abstract and in the last paragraph of the introduction:

PAGE 2: "This model is a 2D representation of 3D cell migration."

2. Page 10, line 190: The authors mention that a strain-dependent Young's modulus can be used to incorporate strain stiffening into the model. This is the only time a Young's modulus is mentioned in the entire manuscript. A constitutive equation relating elastic stresses to strains should be specified in Supplement S2.

The stress tensor consists of a hydrostatic (pressure) and deviatoric part. The calculation of both parts in SPH is added to S2 Text. Here, the bulk modulus K and shear modulus G are required as material properties. As mentioned, they can both be calculated from the Young's modulus and Poisson's ratio of the material.

The fibrillar mechanics, that have been captured by placing nonlinear elastic springs between ECM particles, could in theory be captured by making this Young's modulus strain-dependent. The implementation is however complicated because a change in deviatoric stress is calculated at every time step. The stress that has been build up in previous time steps would therefore have to be corrected every time the local strain changes. We therefore opted to capture the fibrillar mechanics in the model by using nonlinear elastic springs. The following lines and equations were added to S2 Text:

with $\boldsymbol{\sigma}$ the stress tensor and $\boldsymbol{\nabla}_i W_{ij}$ the derivative of the smoothing kernel W. The stress tensor $\boldsymbol{\sigma}$ for an elastic solid consists of the hydrostatic and deviatoric stress:

$$\sigma_i^{\alpha\beta} = -p_i \delta^{\alpha\beta} + S_i^{\alpha\beta}, \tag{3}$$

written in Einstein notation with respect to the coordinate indices α and β, with δ denoting the Kronecker delta, p the hydrostatic pressure and S the deviatoric stress. The hydrostatic pressure is calculated using the equation of state that defines the relationship between pressure and density of a particle:

$$p_i = p_0 + K\left(\left(\frac{\rho_i}{\rho_0}\right) - 1\right), \tag{4}$$

where ρ_0 and p_0 are the initial density and pressure and K is the bulk modulus ($K = \dfrac{E}{3(1-2\nu)}$, with E the Young's modulus and ν the Poisson's ratio) [2]. The deviatoric stress according to Hooke's law reads:

$$S^{\alpha\beta} = 2G\left(\epsilon^{\alpha\beta} - \frac{1}{3}\delta^{\alpha\beta}\epsilon^{\gamma\gamma}\right), \tag{5}$$

with G the shear modulus ($G = \dfrac{E}{2(1+\nu)}$) and ϵ the strain tensor. The Jaumann rate of change of the deviatoric stress is then calculated by:

$$\frac{\mathrm{d}S_i^{\alpha\beta}}{\mathrm{d}t} = 2G\left(\dot{\epsilon}_i^{\alpha\beta} - \frac{1}{3}\delta^{\gamma\gamma}\dot{\epsilon}_i^{\alpha\beta}\right) + S_i^{\alpha\beta}\Omega_i^{\beta\gamma} + \Omega_i^{\alpha\gamma}S_i^{\gamma\beta}, \tag{6}$$

with $\dot{\epsilon}$ the strain rate tensor:

$$\dot{\epsilon}^{\alpha\beta} = \frac{1}{2}\left(\frac{\partial v^\alpha}{\partial x^\beta} + \frac{\partial v^\beta}{\partial x^\alpha}\right) \tag{7}$$

and $\boldsymbol{\Omega}$ the spin tensor:

$$\Omega^{\alpha\beta} = \frac{1}{2}\left(\frac{\partial v^\alpha}{\partial x^\beta} - \frac{\partial v^\beta}{\partial x^\alpha}\right). \tag{8}$$

3. Section Protrusion Dynamics: The introductory paragraph in this section provides a nice overview. However, as one is reading the subsections, it is not clear to the reader that protrusion growth and maturation each occur for a fixed, predetermined amount of time. It is an important assumption of the model that protrusion lifetime is determined only from the adhesion disassembly rate, and it should be made clear early in this section that this is the assumption and that the rates of growth and maturation are fixed. Also, a justification/explanation of *why* these assumptions are made, specifically why growth and maturations rates are fixed, should be provided earlier in the manuscript rather than only at the end of the discussion section.

The fixed growth time and maturation time are indeed assumed to occur for a fixed time and implemented as such. The growth time of a protrusion in a real cell is likely to be regulated by for example actin availability, chemical signaling and the mechanochemical feedback at the protrusive front. Since actin protrusion is captured only by a protrusive force and no mechanical feedback during protrusion growth has been included yet in the model, a growth time has to be predetermined. By setting the same protrusion growth time for all protrusions of a cell, the model allows to investigate the effect of protrusion length (reasonably equivalent to protrusion growth time) on cell migration. In the results section "Stochastic variability in protrusion lifetime regulates cell migration" we have varied this protrusion growth time and investigated the effect on cell migration. We observed that a change in

protrusion growth time had no significant effect on absolute cell migration velocity.

For the maturation phase a fixed maturation time is required due to the way maturation is implemented. First, we have to fix the base of the protrusion during maturation to make sure that maturation depends on the local ECM stiffness and is not affected by contraction of other protrusions and the cell body. This requires the introduction of a maturation phase and we thus have to set a certain time during which the protrusion is maturing. Second, the strength of adhesion and actomyosin contraction depend on the number of maturation steps that occur in a time period. In order to obtain different adhesion and actomyosin contraction strengths for protrusions that sense different local ECM stiffnesses, we have to set the same fixed maturation time for all protrusions. The maturation time of 600s was chosen such that the maturation factor f_{mat} varied between 0 and 1 for the chosen ECM stiffness values. A lower maturation time would result in weaker protrusions, but the ratio in maturation factor for protrusions that sense different local ECM stiffnesses would be preserved. For a real cell, such a maturation phase of course does not exist and is a continuous process.

The choice for a fixed lifetime of these 2 processes is now highlighted and explained in the following sections:
"Protrusion dynamics":
PAGE 13 – LINES 248-249:
"Protrusions grow, *for a prescribed time,* *by weakening the actin cortex and pushing the membrane outwards"*
PAGE 13 – LINES 251-253:
"Next, the protrusion probers the ECM and metures based on the local ECM stiffness, again for a prescribed time (section Protrusion maturation)"
PAGE 13 – LINES 259-260:
"Due to the fixed growth and maturation times, differences in lifetime between protrusions are only caused by adhesion disassembly."
"Protrusion growth and cell-ECM adhesion"
PAGE 16 – LINES 321-326:
"A prescribed growth time is chosen because the factors that regulate protrusion growth, such as chemical signaling, actin availability and mechanochemical feedback at the protrusive front are not included. This also allows to vary the protrusion growth time between cells in order to investigate the effect of protrusion length (which is reasonably equivalent to protrusion growth time) on cell migration."
"Protrusion maturation"
PAGE 17 – 352-354:
"A fixed and equal maturation time is prescribed for all protrusions to ensure that differences in protrusion and adhesion maturation are only the result of the implemented mechanosensing mechanisms."

4. Page 19, line 418-419: Here the absolute migration velocity is described. Is this the best way to measure *velocity*? The term velocity is typically understood as the rate of change of the position along a path, and the authors do calculate a *path velocity* later in the results section of the manuscript. An explanation of why the authors use an absolute migration velocity instead of the path velocity is needed. Is this velocity used in experimental results? If so, a reference should be given. If this choice of velocity is not based on experimental results a justification to its use should be provided.
We agree that the path velocity would be the most obvious velocity to characterize cell migration. This velocity is also commonly used in experimental studies. However, using this velocity can be treacherous with the current model. Due to the 2D representation, the protrusions occupy a significant part of the cell

volume (or actually surface). Protrusions that grow and quickly retract will not displace the cell body, but they will result in a significant change of position of the cell's center of mass and therefore velocity along the cell path (the path moves back and forth in the protrusion growth direction). A cell that forms many short-living protrusions would not displace the cell body, but would still have a large average velocity along the cell path. On the other hand, a cell that moves by forming only a few, but very effective, protrusions, would have a low average migration velocity along the cell path. In order to avoid any misinterpretations, the average absolute migration velocity was analyzed. This is only the result of the start position of the cell at 0 hours and the end position after 6 hours and is not affected by protrusion dynamics. If our model would have been 3D, the relative volume of a protrusion with respect to the cell body would be much lower and the effect of protrusion dynamics on the migration velocity along the cell path would be less. Therefore, we think that an absolute migration velocity is more robust to access the net cell migration . In the study with a prescribed number of simultaneous protrusions, we also show the migration velocity along the cell path (Fig. 11 and 12) in order to illustrate that forming more protrusions can result in more migration along the cell path, but not absolute velocity.
An explanation for this choice of migration velocity calculation is added at the beginning of section "Stochastic variability in protrusion lifetime regulates cell migration":
PAGE 21 – LINES 463-472:
"The absolute migration velocity rather than the migration velocity (off the cell's center of mass) along the cell path is analyzed here because the former velocity is affected less by protrusion dynamics. Due to the 2D representation of 3D cell migration, the area of a protrusion relative to the cell body area is significant. Therefore, protrusions that retract immediately after being formed do not displace the cell body, but result in a significant migration velocity of the cell's center of mass along the cell path. Cell migration along the cell path could therefore be overestimated and be determined by the number of protrusions rather than the efficiency of protrusions to cause a net displacement of the cell body."

5. Following up on comment above: The manuscript would benefit from validating a portion of the model by comparison to experimental results. One suggestion would be to simply reference experimental results which verify that measured absolute migration velocities (or cell path velocities) compare to the numerical results. A stronger comparison would be to compare experimentally measured absolute migration velocities (or, again, cell path velocities – whatever is more appropriate given the experimental set up) for different substrate stiffnesses to the numerical results in Figure 7.
The reported experimental results of 3D cell migration velocity as function of ECM stiffness vary a lot. Migration velocity has been reported to increase with stiffness [Mason2013], decrease with stiffness [Ehrbar2011,Wolf2013], or follow a biphasic response [Fraley2015]. The reason for this variation is that multiple factors simultaneously affect migration velocity. An increase in ECM stiffness can for example be accompanied by an increase in crosslinking and therefore a decrease in pore size of the ECM. Therefore, an increase in ECM stiffness can result in a stronger cell, but at the same time in an increase hindrance for the cell. Because of this large variation in experimental results, it is difficult and risky to compare the stiffness-velocity relationship observed for the model with experimental data. This was already discussed briefly in the "Discussion" section and has now been extended to:
PAGE 34 – LINES 815-823:
"Ehrbar et al. showed that 3D cell migration in a PEG hydrogel is reduced by an increase in gel stiffness [40]. The same effect was observed by Wolf et al. for migration through a collagen gel, while Lang el al. and Fraley et al. observed biphasic responses for migration through a collagen gel with either highest or lowest migration velocity at intermediate ECM stiffnesses [35,42,47]. These observations are different from our observation that cell migration increases with ECM stiffness. An explanation for this is that an increase in ECM stiffness is accompanied by an increase in cross-linking density, which hinders cell migration. In order to migrate through a densely cross-linked gel more ECM degradation is required."

We agree with the reviewer that it is a good idea to compare the observed migration velocities for the model with experimental data. We have looked at the migration velocities along the cell path from 5 references and can see that our absolute migration velocities are realistic for 3D cell migration. The following lines have been added to the second paragraph of section "Stochastic variability in protrusion lifetime regulates cell migration" to include this comparison with experimental results:
PAGE 21 – LINES 472-478:
"The mean absolute migration velocity per stiffness ranges from 3.7-10.2 µm/hr. Experimentally measured 3D cell migration velocities along the cell path, as reported in literature for various ECM and cell types, range from 0-60 µm/hr [10,35,40-42]. Since migration velocities along the cell path are higher than absolute migration velocities, the absolute migration velocities measured here are realistic for 3D cell migration. The observed increase in migration velocity with higher ECM stiffness can be explained from the model implementations.

6. Page 25, lines 569-571: The first two sentences here are confusing and seem to contradict one another. They need to be reworded so the point author is attempting to make is clearer.
The second sentence is changed to:
PAGE 27 – LINES 625-626:
"However, directly changing these protrusion dynamics does not affect cell migration."

7. Figure 11 and last sentence on paragraph p. 28 lines 651-653: Figure 11 is not particularly enlightening as is. Possibly, instead of displacements, colors can correspond to stresses since the applied stresses are a measure of the maturity of the adhesion. The idea is to better illustrate that protrusions in the direction of the fibers are more mature than adhesions perpendicular to the fibers. As it stands currently, this figure does not do much to support the claim on lines 651-653.
We agree that the effect of the ECM on competition between protrusions can be visualized better by showing stresses in the ECM instead of displacements. Since this figure is made for a uniaxial fibrillar ECM, the nonlinear elastic part of the ECM is captured by nonlinear springs. Therefore, in contrast to a homogeneous ECM modeled by viscoelastic NSPH, a (continuum) stress tensor is not calculated for the fibrillar part of the ECM during the simulation. In order to change the figure, we therefore opted to calculate a Cauchy stress tensor for the fibrillar part of the ECM, based on derivation by Cioroianu et al. for an elastic spring network. Finally, a Von Mises stress is calculated from this Cauchy stress tensor and shown in Figure 13 (former Figure 11).
The calculation of the Cauchy stress tensor is added to the manuscript at the end of section "Optimal number of simultaneous protrusions depends on ECM anisotropy":
PAGE 30 – LINES 708-716:

with 2 simultaneous protrusions. This figure shows the Von Mises stress distribution in the uniaxial fibrillar ECM during migration. Since the stress in the nonfibrillar part of the ECM model is negligible compared to the stress in the fibrillar part, the Von Mises stress is calculated only for the fibrillar part. The Von Mises stress is calculated from the Cauchy stress tensor. This Cauchy stress tensor is calculated for each solid ECM particle, based on a derivation by Cioroianu *et al.* for an elastic spring network [43], as:

$$\boldsymbol{\sigma}_i^{c,\alpha\beta} = \sum_{j\in\mathcal{S}\setminus i} \frac{1}{V_i \det\left(\boldsymbol{\mathcal{F}}_{ij}\right)} \|\boldsymbol{F}_{ij}^{\text{fib}}\| \left(\frac{\boldsymbol{x}_{ij}^{\alpha}\boldsymbol{x}_{ij}^{\beta}}{\|\boldsymbol{x}_{ij}\|}\right), \tag{11}$$

written in Einstein notation with respect to the coordinate indices α and β, with $\boldsymbol{\sigma}^c$ the Cauchy stress tensor, $V = \frac{m}{\rho}$ the particle volume and $\boldsymbol{\mathcal{F}}$ the deformation gradient tensor for the spring between particles i and j.

8. Page 30, lines 689-691: There is no evidence in the paper to support the claim "In our model, varying protrusion number, lifetime and length did not affect both the average absolute migration velocity and ***average migration velocity along the cell path*** ". The average migration path velocity is only measured when number of protrusions is varied (not lifetime and length of protrusion). This sentence should be modified to reflect what is supported with results.

The sentence is changed such that "migration velocity along the cell path" is not discussed here. We hope that the reply to comment 4 clarifies why we have chosen to analyze absolute cell migration velocity, although migration velocity along the cell path is mentioned in the experimental paper that we refer to. The aim of this section is to illustrate that changes in both migration velocity and protrusion dynamics do not necessarily imply a causal relation, rather than to directly compare experimental results with our model outcomes. We therefore deem the absolute migration velocity to be a proper model outcome to analyze.

Minor comments:

9. The work most similar to the manuscript is what has been published in reference [11]. However, a major aim of the work in reference [11] is to determine the effective cellprobed stiffness of the ECM, which is entirely different than the aim of the submitted manuscript. The authors should highlight this as it will strengthen the novel contribution they are making with the manuscript.

A sentence is added to mention that the emphasis of this work lies on the development of a method for local stiffness sensing and not the mechanism of 3D cell migration:

PAGE 5 – LINES 68-70:

The main emphasis of their work, however, does not lie on 3D migration mechanisms, but on the development of a method to characterize the local stiffness sensed by a cell in a fibrillar material.

10. Figure 4 caption: Parenthetical should state "(see Eq 7 *and surrounding text* for parameter meanings)."

The suggested text is added to the caption.

11. Figure 7 is difficult to read. It should be broken up into two figures. One option is to put ECM stiffness and cell strength variations in one figure and adhesion initiation rate, disassembly rate, and growth time in another figure.
Figure 7 has been split into two Figures (now Figure 7 and 8) as proposed by the reviewer. The caption and references to the figure in the text have been adapted accordingly.

12. In Figures 7-10, an explanation of the horizontal bars at the top of each panel is needed.
The following sentence is added to these captions:
"Horizontal bars indicate a significant difference between outcome parameter values of two groups of 12 simulations, calculated by means of the unpaired two-sample t-test."

13. Figure 8 is also difficult to read. Break up into two figures. Part (A) can go into one figure while parts (B)-(D) can go into another.
Figure 8 has been split into two Figures (now Figure 9 and 10) as proposed by the reviewer. The caption and references to the figure in the text have been adapted accordingly.

14. Figure 9 caption and Figure 10 caption: "Time lag" needs to be defined.
Time lag τ is now mentioned in the caption and the MSD formula is added to the main text just above the figure:
PAGE 28 – LINES 672-673:
"mean squared displacements (MSDs) as function of time lag τ (steeper slope, with $MSD(\tau) = \langle[\mathbf{x}(t+\tau)-\mathbf{x}(t)]^2\rangle$ and time t)"

15. Page 30, line 693-695: Looking at Figure 8 B-D, visually, there is a difference in average migration velocities between force dependent and independent adhesion disassembly. Rather than stating that no difference in migration velocity was observed, it would be better to state the differences in migration velocity are not statistically significant.
PAGE 32 – LINE 756:
"no difference" is changed to "no significant difference"

16. S1 Text, line immediately after equation (2): should be unit vector from i to j (not j to i, as stated).
We discovered a mistake in equation (2) of S1 Text. The equation is corrected to "e_i-e_j / |e_i-e_j|", such that the statement "unit vector from j to I" is correct.
The usage of $v_{ij} = v_i - v_j$ is common practice in SPH and is used throughout the paper as stated at the beginning of the Methods section in section "Deformable cell model".

17. S1 Text, lines immediately after equations (5) and (6): "line segment z" needs to be defined.
Two sentences are extended to clarify that the line segment z is a line connecting two neighboring boundary particles and is part of the cell boundary:
"with A the cell area, A the resting cell area, k_A the area constraint constant and n_z the outward normal to line segment z that connects neighboring cell boundary particles"*
"In order to prevent the cell boundary from penetrating itself, a repulsive Hertz-like force is applied to boundary particles that approach a line segment connecting two other neighboring boundary particles of the same cell"

Reviewer 2

It was a pleasure to review "The role of actin protrusion dynamics in cell migration through a degradable viscoelastic extracellular matrix: Insights from a computational model" by Heck et al. My background is in the biology of cell migration, so I have reviewed the manuscript from that perspective and limit my comments to the biological aspects of the study and the accessibility of the study to experimental biologists, including the plausibility of the biological assumptions and the significance & novelty of the conclusions. I make no comment on the mathematical or computational aspects of the study.

This manuscript tackles the important question of the regulation of 3D cell migration which is an important element of many physiological and pathological processes including wound healing, the immune response and cancer metastasis. This is a question that is challenging, although not impossible, to address experimentally and so would benefit from a computational model that allows rapid interrogation of hypotheses. The computational model is very well described and clear to biologists. It makes plausible assumptions about the behaviour of cells, cellular protrusions and the ECM and the model that is generated allows for significant and novel conclusions about the behaviour of cells in three dimensional environments to be reached. These conclusions have the ability to be experimentally verified making them useful for cell biologists working in this field.

Overall, I thought this was a well written study that will be of broad interest to cell biologists. Below I make some minor comments that could be addressed to strengthen the manuscript.

We thank the reviewer for appreciation of the work and highly prize the comments from a biological perspective.

1. The model in this study represents a model of generic cell migration and the manuscript may benefit from discussion about how this might model some specific example of cell migration, for example cancer cells during ECM invasion as a part of metastasis or neutrophils responding to chemokines during infection. These can be processes that are very different in their regulation, with different scope for ECM degradation, different requirements for chemotaxis and can result in cells transitioning through different ECM environments. This is also a model of individual cell migration through ECM and I wonder if a similar approach would also model collective cell migration which is often seen during wound healing and some types of cancer metastasis.

The current model is indeed a generic model of single cell migration rather than a model that is tuned for a specific situation. The model is therefore tunable and adaptable in order to investigate cell migration in specific situations. The main limitation of the current model is the lack of structural information in the ECM model. The ECM model is able to describe complex coarse-grained ECM mechanics, but does not capture ECM structures such as individual fibers and micrometer-sized pores. The latter means that ECM degradation is required during cell migration in the current model. Therefore, the model is not able to describe amoeboid migration as is seen for example for neutrophils. Different cell types that use mesenchymal migration can be captured and modeled in detail by tuning the subcellular processes of the model with experiments. In addition, chemotaxis can be added by including intracellular signaling in the model that is regulated by external chemokine gradients. Modeling of multicellular systems would be possible as well, although limited to a few cells due to computational costs. It would be very interesting to see the effect of ECM degradation on collective migration. Migration in cell strands might be a solely the result of hindrance by the ECM, which forces cells to follow degraded tunnels created in the ECM by leading migrating cells. A paragraph has been added at the end of the Discussion section to elaborate on these topics:

PAGE 35 – LINES 847-867:

“The current model could be adapted and extended to investigate various cases of both single cell migration and collective migration. An important condition would be that the studied migration mode should be based on similar mechanisms as those described here, namely protrusion formation, cell-ECM adhesion, actomyosin contraction and ECM degradation. Therefore, amoeboid migration, which is observed for example for neutrophils during inflammation, would be less suitable to investigate with our model, mainly because ECM degradation is required for migration due to the lack of micrometer-sized pores in the ECM model. In order to investigate migration for a specific cell type and extracellular environment, tuning of the model based on experiments will be important. Properties such as protrusion dynamics, adhesion lifetimes and ECM degradation can be observed through optical microscopy, while 3D traction force microscopy is an interesting technique to tune the contractile strength of the cell model. Depending on the cell type, regulation of subcellular processes by external chemical signals, resulting in chemotaxis, might also be an indispensable for understanding migration. Intracellular signaling, regulated by external chemical signals, could be modeled by means of partial differential equations at the cell domain and would be a logical future extension of the model. Finally, collective cell migration could be modeled with the current model by adding cell-cell adhesion and intercellular signaling. It would be particularly interesting to investigate the effect of tunnel formation in the ECM on migration of strands of cells as seen in for example angiogenesis and collective cancer cell metastasis.”

2. Throughout the manuscript the authors generally refer to “cell protrusions”. Filopodia are mentioned several times but to me the protrusions as described in the manuscript, particularly their involvement in ECM degradation, may better resemble invadopodia or podosomes (depending on whether the cells in questions are cancer cells or normal cells). Some brief mention or discussion of these invasive types of protrusions would be helpful.

Filopodia are mentioned only when discussing the model of Kim et al. and when proposing future improvements of the model, by adding local ECM sensors that regulate protrusion formation. It is indeed true that the protrusions captured by the model do not represent filopodia. In order to clarify this, the first paragraph of the introduction is extended to elaborate on different migration modes and protrusion types. Since the use of different terms for cellular protrusions are sometimes somewhat inconsistent in literature, we prefer the use of actin protrusions throughout the paper. We hope that the extension clarifies that our model focusses on long actin-rich protrusions used for mesenchymal cell migration. The following sentences were added to the introduction:

PAGE 4 – LINES 21-23:

“This fast migration mode, which is characterized by weak cell-ECM adhesion and low ECM degradation, is called amoeboid migration.”

PAGE 4 – LINES 26-38:

“The formation and contraction of these protrusions, in combination with strong cell-ECM adhesion and ECM degradation, results in a migration mode that is called mesenchymal migration. Various protrusions have been reported in both migration modes that range in size, function and protrusion mechanism [6-8]. Filopodia are short needle-like protrusions that are used to sense local chemical and mechanical cues, but are not involved displacement of the cell body. Pseudopodia are longer cylindrical protrusions that form by actin polymerization, similar to lamellipodia in 2D, and are able to displace the cell body by actomyosin contraction. Lobopodia are also longer cylindrical protrusions, but like blebs they are formed by membrane extension due to intracellular pressure. Finally, invadopodia are actin-rich protrusions that have been reported to be used by cancer cells to breach and invade the basement membrane [9] Here, we focus on 3D migration by formation and contraction of long, actin-rich protrusions.”

3. There is a lack of experimental verification of the model. There is some discussion of the Fraley paper

that supports some of the model conclusions, but other conclusions lack experimental support. I'm not suggesting that experimental support is necessary in this study and indeed I feel it is both extremely challenging to do and well outside the scope of the study but perhaps some discussion of future experimental steps would be helpful.
Based on this comment, and the comment from another reviewer, we have added some lines to the results section to compare our absolute migration velocities with experimentally measured migration velocities. Here, the response to reviewer 1 is copied:

The reported experimental results of 3D cell migration velocity as function of ECM stiffness vary a lot. Migration velocity has been reported to increase with stiffness [Mason2013], decrease with stiffness [Ehrbar2011,Wolf2013], or follow a biphasic response [Fraley2015]. The reason for this variation is that multiple factors simultaneously affect migration velocity. An increase in ECM stiffness can for example be accompanied by an increase in crosslinking and therefore a decrease in pore size of the ECM. Therefore, an increase in ECM stiffness can result in a stronger cell, but at the same time in an increase hindrance for the cell. Because of this large variation in experimental results, it is difficult and risky to compare the stiffness-velocity relationship observed for the model with experimental data. This was already discussed briefly in the "Discussion" section and has now been extended to:
PAGE 34 – LINES 815-823:
"Ehrbar et al. showed that 3D cell migration in a PEG hydrogel is reduced by an increase in gel stiffness [40]. The same effect was observed by Wolf et al. for migration through a collagen gel, while Lang el al. and Fraley et al. observed biphasic responses for migration through a collagen gel with either highest or lowest migration velocity at intermediate ECM stiffnesses [35,42,47]. These observations are different from our observation that cell migration increases with ECM stiffness. An explanation for this is that an increase in ECM stiffness is accompanied by an increase in cross-linking density, which hinders cell migration. In order to migrate through a densely cross-linked gel more ECM degradation is required."

We agree with the reviewer that it is a good idea to compare the observed migration velocities for the model with experimental data. We have looked at the migration velocities along the cell path from 5 references and can see that our absolute migration velocities are realistic for 3D cell migration. The following lines have been added to the second paragraph of section "Stochastic variability in protrusion lifetime regulates cell migration" to include this comparison with experimental results:
PAGE 21 – LINES 472-478:
"The mean absolute migration velocity per stiffness ranges from 3.7-10.2 µm/hr. Experimentally measured 3D cell migration velocities along the cell path, as reported in literature for various ECM and cell types, range from 0-60 µm/hr [10,35,40-42]. Since migration velocities along the cell path are higher than absolute migration velocities, the absolute migration velocities measured here are realistic for 3D cell migration. The observed increase in migration velocity with higher ECM stiffness can be explained from the model implementations.

Besides, the fact that a tighter coupling with experiments is required is added at the end of the "discussion" section. It is mentioned that experiments should be performed that allow to vary or tune specific components of the cell or ECM, which should enable a better understanding of 3D cell migration:
PAGE 36 – LINES 882-885:
"Finally, a tighter coupling with experiments is required. Ideally these experiments should allow to vary or tune processes that are captured by model parameters, such as ECM stiffness, ECM degradability and protrusion dynamics, to further increase our understanding of 3D cell migration."

4. Some of the model parameters shown in Table 1 are well chosen and supported by the literature but

some – such as cortex stiffness, actomyosin force, maturation time etc – are not and are described as trial runs or model setup. How were these parameters chosen and how well do they reflect biological reality? While I imagine some of these parameters have not been measured in 3D systems, there may be quantification of them in 2D systems that may at least be a reasonable starting point.

We have reviewed the parameters in the tables. Many of these parameters are chosen to ensure numerical stability and do not necessarily represent experimentally observed cell physics. For example, cortex bending rigidity and area conservation are assumed negligible, and therefore included in the model only to prevent membrane folding and excessive cell shrinking. Below, the parameters without reference are discussed:

- *All parameters with "model setup[2]" as reference in the table are set based on the chosen resolution for the cell and ECM model. For example, the number of cell boundary particles that are part of a protrusion are chosen based on the particle distance and the number of particles in the cell model. As a second example, the adhesion length of 5 µm is chosen to make sure that the adhesion force is applied to a point far enough in the ECM to ensure that the force is distributed over sufficiently larger number of ECM particles (using the smoothing kernel). Therefore, the adhesion length parameter does not represent a physical distance between actin inside the cell and ECM molecules outside the cell (which would be a few hundred nanometer), but is a parameter set to ensure numerical functionality. We have therefore opted not to make changes in the tables to parameters with "model setup[2]" as reference*
- *A reference is added for the cortex stiffness (Cartagena-Rivera 2016). The chosen actin cortex stiffness ($2.8x10^{-3}$ N/m) is obtained by assuming a cortex thickness of 0.4 µm and Young's modulus of 6 kPa. The latter falls in the range of cortex stiffnesses reported in literature, which vary from Pa to tens of kPa. The reference has been added to Table 1 and the sentence*
  "($2.8x10^{-3}$ N/m, assuming a cortex thickness d_c=0. 4 µm and Young's modulus E_c=6 kPa and calculated with $k_s = 2E_cd_c/\sqrt{3}$)"
  is added to S1 Text.
  It is still a question what the importance is of cortex stiffness for migration in the current model. First, since a weak actomyosin contraction is applied to the cell body, contraction of protrusions will anyway result in displacement of the cell body. Second, the actin cortex has been reported to behave as an elastic solid only at the timescale of seconds. At larger timescales, due to turnover of actin and actin-binding proteins, stresses dissipate and the cortex is reported to behave as a viscous fluid. We therefore think that tension in the cortex due to actomyosin contraction plays a more important role in the model than actin cortex stiffness. More simulations could be performed in the future to analyze this.
- *The chosen cortex viscosity lies at the higher end of values used and/or obtained for other computational models: $1.5x10^{-6}$ Ns/m to $6x10^{-3}$ Ns/m (Kim 2014, Biophys J; Odenthal 2013, PLOS Comp Biol; Kim 2017, PNAS).*
- *A reference is added for the cortex bending rigidity (Cartagena-Rivera 2016) (see cortex stiffness). The following sentence is added to S1 Text:*
  "($2x10^{-15}$ Nm, which is significantly higher that calculated with the formula $k_b = E_cd_c^3/12(1-v_c^2)$, with v_c=0.45 the Poisson's ratio of the cortex, in order to ensure that cortex folding is prevented)"
  Although a value is set for this parameter, it is used merely to stabilize the simulation (by preventing folding at high angles) and will not affect cellular behavior.
- *k_A, k_{rep} and d_{rep}, are parameters that are set to prevent cell distortions, but do have a physical foundation.*
- *γ_{liquid}, the liquid drag force constant, is set a few orders of magnitude higher than expected for extracellular fluid in order to ensure numerical stability. The value is chosen such that the actin*

polymerization force, applied to cell boundary particles during protrusion growth, results in a realistic protrusion growth speed as mentioned in the text.

- *The actin cortex stiffness during protrusion growth is reduced with three order of magnitude in order to allow actin polymerization forces, which are low compared to actomyosin contraction forces, to push the weakened cortex outwards to form a protrusion. This has been mentioned in the text.*
- *Protrusion deflection rate r_{defl} is used to rotate protrusion growth away from the solid ECM. For a real cell, growth direction depends on local microscale ECM characteristics. This parameter can therefore not be validated with experiments.*
- *The adhesion stiffness is chosen significantly stiffer than the ECM to ensure that mechanosensing depends only on ECM mechanics. Two lines are added to section "Protrusion growth and cell-ECM mechanics" to clarify this:*
  PAGE 16 – LINE 327:
  ", with stiffness k_{ad},"
  PAGE 16 – LINES 329-331:
  "The adhesion is chosen to be significantly stiffer than the ECM such that mechanosensing by the protrusion depends solely on ECM mechanics (see section Protrusion maturation)."
- *T_{prot}, T_{mat} and T_{finish} are chosen to ensure realistic protrusion lengths, proper mechanosensing time and retraction of a protrusion in case it did not manage to displace the cell body before adhesion disassembly. For a real cell, separate phases of protrusion growth, maturation and retraction do not exist and likely happen simultaneously. These parameters can therefore not be validated by experiments and were instead chosen, based on trial simulations, to ensure realistic cell behavior.*
- *Two references are added for the reference actomyosin contraction force F_{am}*
- *Finally, as mentioned in the manuscript, degradation rates were chosen higher than would be expected to prevent numerical instabilities from occurring. In the future, these parameters could be tuned based on experimental data.*

5. Just a comment rather than anything else but the geometry or architecture of the ECM in vivo is often more ordered or structured than that found in many in vitro systems (such as collagen gels) which often have chaotic and disorganised fibre structure. The model in this manuscript is modelling in vitro environments but could easily be adapted to model in vivo ECM environments with different ECM architecture. It would be interesting to see if that changes the behaviour of the cells.

We agree that cases of cell migration in an in vivo environment with specific ECM characteristics would be a nice future step. The model has the capability to capture mechanical anisotropy by favoring fiber direction as was shown in the manuscript. In addition, stiffness gradients, rigid obstacles and specific ECM shapes could be implemented easily. The following lines have been added to the discussion to further elaborate on this:
PAGE 34 – LINES 805-814:
"The current ECM model could be used to investigate the effect of structured ECM architectures on 3D cell migration. For example stiffness gradients, rigid obstacles, specific ECM geometries and mechanical anisotropy by favoring fiber direction can already be implemented easily. Currently, the main limitation of the model is that springs in the ECM model do not represent individual collagen fibers, but are a coarse-grained representation of the nonlinear mechanical behavior. Therefore, the cell does not attach to and pull on individual collagen fibers and the ECM does not present a porous structure for the cell to

migrate through, which This makes it challenging to distinguish between an ordered and disordered fiber structure."

6. A very minor point – on page 12 the sentence "Protrusions grow by weakening the actin cortex and pushing the membrane outwards" initially read as if it was describing bleb formation. I assume – as is made clear on page 13 – that "pushing the membrane outwards" is accomplished by actin filament polymerisation rather than through hydrostatic pressure but this could be made clearer.

Pushing out of the membrane indeed is the result of actin polymerization and not internal pressure. The sentence is adapted to clarify this by adding:

PAGE 13 – LINE 250:

"through directed actin polymerization"

Reviewer 3
In this work, authors present an hybrid approach combining an agent-based mechanical cell model and a meshless Lagrangian particle-based degradable viscoelastic ECM model in order to simulate 3D cell migration.

In my opinion, the most interesting contribution of this work is the novel numerical approach that has been proposed. In fact, it is really novel and innovative approach to simulate 3D cell migration by means of this hybrid approach. However, in the case of the model, there are some assumptions and simplifications that should be commented and better justified. Three main assumptions are my main concern in this work:
We thank the reviewer for his appreciation of the methodological novelty.

• In this work, it is assumed that cell polarization is well defined, clearly fixing the front and the rear of the cell. However, the polarization of the cell during migration in 3D is not well defined, being multiple, because many protrusions are competing between them to define their movement.
Polarization of the cell in our model is indeed implemented by distinguishing a front half and rear half of the cell. The polarization direction is set initially in the direction of the first protrusion and later rotates towards the average direction of mature adhesions. In this way, when competition between protrusions favors a protrusion (because of a lower adhesion disassembly rate or because neighboring protrusions rupture due to a too high force), the polarization direction of the cell rotates towards this "winning" protrusion.

Although a polarization direction is defined for the cell, this does not mean that the cell migrates in the polarization direction. Protrusions are allowed to form at the entire front half of the cell and grow in the direction away from the center of mass. Therefore, at any time protrusions can grow in a range of angles of 180° (from 90° to the left up to 90° to the right). Since the model normally forms 2 to 3 simultaneous protrusions, there is always competition ongoing between "protruding fronts". The more favorable protrusion is more likely to exist longer and will therefore pull the cell in its direction and at the same time rotate the polarization in its direction. This change in polarization direction changes the possible growth direction of future protrusions. Therefore, although a single polarization direction is set, there is always a competition ongoing between multiple protrusions to determine the direction of cell migration, resulting in a typical random walk migration.

The following lines have been added at the end of section "Protrusion initiation and cell polarization":
"It is important to note that, although a single polarization direction is defined, multiple protrusions will be initialized at the front half of the cell and will grow in different directions. Therefore, competition between multiple protrusions, that affect cellular polarization, is captured in this model."

• The cell nucleus has not been considered in the model and however its role is crucial in 3D migration. I think it is a strong simplification in the model for simulating 3D migration.
We agree that the role of the nucleus can be very important in 3D cell migration as it can hamper movement of the cell through narrow spaces in the ECM. In the current model, as is highlighted in the discussion, degradation of the ECM is quick compared to real hydrogels. So although a degraded tunnel must be created for the cell to migrate, no situations are captured with the model yet in which the cell has to squeeze through a narrow opening. Addition of a rigid nucleus is therefore not expected to affect the results presented in this manuscript. If in future cases we would want to make cell migration more dependent on cellular deformation by simulating migration of the cell through an ECM with narrow openings, inclusion of a nucleus is indeed indispensable.

The following sentence is added to the end of the discussion to highlight the importance of including a deformable nucleus to the cell model:
"Besides, the addition of a deformable nucleus is necessary if cell migration is hindered due to slower ECM degradation, forcing the cell to deform strongly in order to move through narrow openings in the ECM."

• Although it is true that there are not differences from a concept point of view, cell movement is being really modeled as a 2D phenomenon. In my opinion, it is a limitation when you have to interpret results from in-vitro experiments, in which movement is clearly 3D.
This is indeed an important difference that has to be taken into account when comparing the results with experimental data. Since the implemented methods would be implementable similarly for a fully 3D model, we believe that our main results would remain qualitatively the same. Thus, the correlations between simulation readouts as shown in Figure S2 and qualitative observations made throughout the manuscript would be similar. The largest difference would probably be observed quantitatively for the migration velocity. A cell in a 2D plane model is not in contact with the ECM above and below that plane, and might therefore be hindered less while migrating through the ECM. The 2D cell is also more likely to rotate toward an area that has already been degraded before. As explained in the discussion, degradation is fast in the current model, which reduces the hindrance of migration by the ECM. The current model might therefore migrate with a similar velocity as a fully 3D model. If in the future simulations with a significantly slower degradation could be performed, hindrance would become more important and a larger difference between 2D and 3D migration velocities might be observed.
The following sentences are added to the fourth paragraph of the discussion section to emphasize the shortcomings of a 2D representation of 3D migration:
"First, the model is a 2D representation of 3D migration. This makes it more difficult to compare migration paths and velocities with 3D cell migration experiments. Besides, this affects the hindrance experienced by the cell model due to the surrounding ECM because for a 2D model the cell is only in contact with the ECM in the considered plane."

Some other assumptions also require further analysis or justification:

• There are some works that indicate that the main component of protrusions is not actin, but tubulin (Fraley et al, 2012).
The role of microtubules in both 2D and 3D cell migration has been studied and discussed in various journal papers. The common perception is that microtubules play an important role in migration and are indispensable for 3D cell migration, especially for the regulation of adhesions and protrusions. Microtubules are involved in Rho GTPase signaling and transport of adhesion receptors to specific locations in the cell (Bouchet 2017). Disruption of microtubules through depolymerization results in an over-activation of Rho-signaling and consequently excessive traction, which limits the possibility for long protrusions to form (Martins 2012). However, the mechanical process of protrusion formation and contraction is driven by to actin polymerization and actomyosin contraction, and not by microtubule polymerization and depolymerization. The fact that the model could be extended by adding chemical signaling, ass stated at the end of the discussion section, could thus also include modeling the effect of microtubules on 3D cell migration. We are however convinced that actin protrusion and actomyosin contraction are the main drivers of protrusion-based cell migration, while microtubules can be regarded as modulators. Microtubules have therefore not been included in the current model

• The assumption that the number of contraction steps required to reach a threshold force is highly dependent on the matrix rigidity could be controversial, because in the experimental work of Mitrossilis et al (2009, PNAS), they measured that the time required for achieving the threshold force was independent of this stiffness.

In the study of Mitrossilis et al. traction force was measured for an entire cell and it was observed that the generated force reached a plateau after approximately 10 minutes, independent of the flexible glass plate stiffness. However, the applied force did scale with stiffness, resulting in a larger plateau force for a stiffer glass plate. The increase of force with time thus also scaled with stiffness. In our manuscript, the force equilibrium at which actomyosin contraction and the resisting force due to ECM deformation are balanced is used to determine the moment at which both actomyosin and the adhesion mature at the scale of a single protrusion. Wolfenson et al. demonstrated that these force equilibriums are reached in a few seconds instead of minutes. The process of increase in traction force as described by Mitrossilis et al. is thus the effect of many maturation steps at a subcellular scale. If we look at the maturation steps for a protrusion in a soft or stiff ECM in our model (Figure 3F), then these force equilibriums are reached faster for a stiff ECM, thus resulting in more maturation steps taking place in the same time frame. This thus means that the increase in contraction force in our model scales linearly with the substrate stiffness, which is in agreement with the observation by Mitrossilis et al. The plateau that they observed defines the saturation of the force applied by the cell. At equilibrium, the cell thus applies a higher force on a stiffer glass plate. Since we chose a fixed maturation time (of 10 minutes) in our model, we observe the same scaling of contraction force with ECM stiffness at the end of the maturation phase.

• In this work, it is assumed that cells are able to generate more force in the direction of the protrusions in function of the matrix stiffness. Have you considered that this force can saturate? Please, see again Mitrossilis et al (2009, PNAS).

The number of maturation steps is limited to 10 in the current model. The ECM stiffnesses and maturation time are chosen such that this plateau is not easily reached and the cell strength increases with increasing ECM stiffness. If the current cell model would be placed in an ECM with higher stiffness, it would reach the maximum number of maturation steps within the maturation time, and thus the cell would not become stronger. The following lines have been added to section "Protrusion maturation" to clarify this:

"A maximum of 10 maturation steps, equivalent to f_{mat}, is allowed in the model. Therefore, the protrusion strength saturates in very stiff ECMs."

• It is not clear for me how the model is able to simulate the porosity of the extracellular matrix, above all in the case of fibrillar hydrogels like collagen gels.

The current ECM model is a coarse-grained mechanical description of the material. It does not capture structural properties of the ECM such as individual fibers or pores. The ECM is thus represented as a continuous, nanoporous ECM, with or without global fibrillar mechanics. The following sentence is added to section "Extracellular matrix model" to clarify this:

"Therefore, the mechanics of a fibrillar ECM is captured, but structural properties such as individual fibers and pores are not included."

• The degradation of the ECM is not clear how has been simulated. In one part of the paper, it is indicated that "ECM particles close to the protrusion tip or the cell body can be degraded with a chosen degradation rate" and in another one is indicated: "Wolf et al demonstrated that proteolysis of collagen fibers does not take place at the protrusion tip, but rather at the cell body where sterically impeding fibers are targeted". Please, revise carefully the paper to avoid this misunderstanding.

Degradation at the protrusion tip is allowed only during protrusion growth. Since the ECM is assumed to be continuous, and thus without porous openings that would allow the protrusions to grow into, degradation is required to create space for the protrusion to grow. After protrusion formation, degradation is allowed only at the cell body in order to meet the experimental observation by Wolf et al. Two sentences are added to section "Protrusion growth and cell-ECM adhesion":
"Degradation of the ECM close to the protrusion tip is only allowed during protrusion growth. Afterwards, ECM degradation only occurs close to the cell body as will be explained in section ECM degradation."
and one sentence to section "ECM degradation":
"ECM degradation at the protrusion tip is only allowed during protrusion growth."
to clarify that ECM degradation is only allowed at the protrusion tip during protrusion growth.

• Adhesions are assumed to rupture at high load, but adhesions also fail at low load. In fact, they are normally simulated by a catch-bond law (see Escribano et al, 2018, BMMB).
Adhesion disassembly in the model is force-dependent as defined by equation (7) and shown in Figure 4. Adhesions are assumed to rupture at high force, stabilize with intermediate force and disassemble quickly at low force. This is in agreement with the combined catch bond and slip bond mentioned by Escribano et al.

The main difference between both implementations is the sharp threshold for adhesion rupturing in our model. The protrusion of a real cell would have multiple cell-ECM adhesions. Disassembly of one adhesion would increase the force that is experienced by the surrounding adhesions, and therefore increase the probability of disassembly for these adhesions. This could result in a chain reaction of adhesion disassembly. Since in our model only one adhesion is formed per protrusion, a sudden increase in adhesion disassembly rate above the threshold rupture force has been implemented.

Finally, the predicted results about 3D durotaxis are a direct consequence of the considered assumptions. However, there is much debate in the bibliography whether or not 3D durotaxis really exists.
Durotaxis is the directed migration of a cell along an ECM stiffness gradient. In this manuscript we do not use a stiffness gradient, but an anisotropic fibrillar distribution (preferred fiber direction), resulting in an ECM with different stiffnesses in different directions. Cell migration in the model is directed towards the direction of highest ECM stiffness.

We do not agree that this directed migration is solely due to the assumptions underlying the model. Protrusions that sense a stiffer ECM stiffness in the model mature more and therefore apply a larger contraction force. However, just the application of more force in the fiber direction does not mean that the cell will only migrate along this direction. These stronger protrusions would contract faster than protrusions formed in other directions, but all protrusions can still displace the cell body to the same amount. Migration would therefore be slightly directed towards the fiber direction, but would not be as directed as we observed in our simulations. The competition between protrusions, which results in rupture of the adhesion of weaker protrusions, is the main mechanism in the model that allows the cell to favor a direction of protrusion contraction and thus migrate in a much more directed manner. Therefore, we think that directed migration in our model is an outcome based on subcellular processes, rather than a direct consequence of the assumptions made.

Finally, 3D cell migration through a fibrillar ECM is clearly more complex than sensing of a stiffness gradient. Mechanosensing by protrusions, as is the case in our model, is dependent on the local ECM

stiffness. The fact that cells pull on a single fiber means that the stiffness sensed is highly dependent on local fiber orientation, crosslinking and degradation and not only on global stiffness. This local stiffness sensing has been discussed and investigated computationally (Kim 2017, PNAS). Besides, an increase in fibrillar ECM stiffness is often accompanied by an increase in fibrillary density and crosslinking. Therefore, even though cells would make stronger protrusions in a stiffer ECM, migration could be hindered due to a smaller pore size of the material. Durotaxis is therefore likely to be less effective in 3D compared to 2D cell migration. In our model, an increase in ECM stiffness or fiber stiffness is not accompanied by increased hindrance of the cell as mentioned in the discussion section. This could explain why migration in our model, due to mechanical anisotropy of the ECM, is more directed than observed in 3D cell migration experiments.

The lack of steric hindrance has been discussed in the discussion section. The following sentence is added to emphasize that the response of a cell to the ECM mechanics and anisotropy should be investigated again when a more realistic hindrance of cell migration by the ECM would be captured in the future:
"In the future, it would be interesting to investigate if the observed responses to ECM stiffness and anisotropy still hold when a more realistic hindrance of cell migration would be captured."
