# Supplementary material for: The role of actin protrusion dynamics in cell migration through a degradable viscoelastic extracellular matrix: Insights from a computational model
Source: PLoS Comput Biol. 2020 Jan 13;16(1):e1007250. doi: 10.1371/journal.pcbi.1007250 (PMC6980736; doi:10.1371/journal.pcbi.1007250)
Supplement: Attachment — Submitted filename: Review of REVISION PCOMPBIOL-D-19-01125_R1.pdf [file pcbi.1007250.s014.pdf]

Review of REVISION PCOMPBIOL-D-19-01125_R1

The role of actin protrusion dynamics in cell migration through a degradable viscoelastic extracellular matrix: Insights from a computational model

Authors: *Tommy Heck, Diego Arango Vargas, Bart Smeets, Herman Ramon, Paul Van Liedekerke and Hans Van Oosterwyck*.

The authors have addressed the concerns of all reviewers and have written a very nice paper in which they describe a novel and interesting modeling technique and use this technique to better understand how protrusion numbers and dynamics affects motility and interaction with the ECM. As I mentioned in my original review, the model and technique presented in this manuscript has various extensions (which are, due to revisions, now better highlighted in the discussion) that can be used for further study of cell-ECM interactions. I feel that the paper should be accepted. However, there are two very minor issues and one clarification that I feel the authors should address before the paper is published.

1) There is a typo in equation (1) of the S2 Text: F_i^P should be F_i^σ.

2) The force F^{mat} on panel D of Figure 3 is very difficult to see in red. Please change the color so that it is clearer that this panel refers to the maturations step.

3) Is equation (10) on page 28/43 correct? If $A_{cell} > A_{cell,0}$, then given equation 10 indicates that the magnitude of the protrusion force will be scaled up not down. The authors state that scaling the protrusion force should mimic the *reduction* of available actin, which should *decrease* the protrusion force. Either the equation needs to be corrected or how the scaling affects the protrusion force should be clarified.
