# Supplementary material for: The role of actin protrusion dynamics in cell migration through a degradable viscoelastic extracellular matrix: Insights from a computational model
Source: PLoS Comput Biol. 2020 Jan 13;16(1):e1007250. doi: 10.1371/journal.pcbi.1007250 (PMC6980736; doi:10.1371/journal.pcbi.1007250)
Supplement: Attachment — Submitted filename: response_to_reviewer1.pdf [file pcbi.1007250.s015.pdf]

*We thank the reviewer for noticing some minor mistakes.*

1) There is a typo in equation (1) of the S2 Text: $F^p$ should be $F^\sigma$.
*The symbol in equation (1) is changed*

2) The force $F^{mat}$ on panel D of Figure 3 is very difficult to see in red. Please change the color so that it is clearer that this panel refers to the maturations step.
*The color of the force and the accompanying symbol is changed to yellow in order to make it clearer.*

3) Is equation (10) on page 28/43 correct? If $A_{cell} > A_{0,cell}$, . , then given equation 10 indicates that the magnitude of the protrusion force will be scaled up not down. The authors state that scaling the protrusion force should mimic the *reduction* of available actin, which should *decrease* the protrusion force. Either the equation needs to be corrected or how the scaling affects the protrusion force should be clarified.
*The ratio $A_{cell}/A_{0,cell}$ was indeed wrong in the equation. The accompanying text is correct, meaning that the protrusion force decrease with an increase in cell area. The equation is corrected.*
